# Transcriptome and Metabolome Analysis of *BmFAMeT6* Overexpression in *Bombyx mori*

**DOI:** 10.3390/genes15101261

**Published:** 2024-09-27

**Authors:** Yang Yu, Tian Li, Ping Chen

**Affiliations:** 1 College of Sericulture, Textile and Biomass Sciences, Southwest University, Chongqing 400715, China; yy18223722377@163.com; 2 Drug Discovery Research Center, Southwest Medical University, Luzhou 646099, China; litianforever@163.com

**Keywords:** *Bombyx mori*, *BmFAMeT6*, growth and development

## Abstract

**Background/Objectives:** The gene-encoding farnesyl diphosphate O-methyltransferase 6 (FAMeT 6) is a member of the farnesyl diphosphate O-methyltransferase family. Our previous studies have demonstrated its influence on juvenile hormone levels in third instar silkworm larvae. **Methods:** we utilized transcriptomic and metabolomic techniques to investigate the changes in third instar larvae at 0, 12, and 24 h following *BmFAMeT6* overexpression. **Results:** (1) The differentially expressed homologous genes were enriched in detoxification-related pathways at all three time points. (2) Transcription factor analysis of DEGs indicated a predominant presence of ZF-C2H2. (3) The metabolite-related network suggested that *BmFAMeT6* may influence the metabolism of silkworm larvae through the ABC transporters, purine metabolism, and tyrosine metabolism pathways. (4) The differential gene count, differential metabolite count, and types of metabolites at the three time points indicated a shift in the regulatory focus within the larvae as time progresses, with the inflection point of regulation occurring at the third instar larval stage, 12 h. **Conclusion:** In summary, our research indicates that the regulatory role of *BmFAMeT6* occurs within the context of the domestic silkworm’s own growth and development regulation.

## 1. Introduction

The domestic silkworm, derived from its wild counterpart, serves as a crucial economic insect and a representative organism within the *Lepidoptera* order. Investigating growth and development regulation during the silkworm larval stage not only contributes to elucidating the molecular mechanisms underlying this process but also yields vital insights for manipulating silkworm development artificially and controlling agricultural and forest pests biologically. The epidermis of an insect is covered by an exoskeleton consisting of three layers of epidermis, as the exoskeleton is rigid, insects must undergo a process called “molting” to shed their exoskeleton and facilitate growth [1]. The larval stage of the silkworm is characterized by multiple moltings that facilitate a complex process of growth and development. Silkworm molting is regulated by two principal hormones: juvenile hormone (JH) and ecdysteroid (ecdysone). The active form of ecdysone, 20-hydroxyecdysone, produced by a P450 enzyme reaction, induces the molting process by binding to a heterodimeric nuclear receptor [2]. Different types of JH exist in insects, and JH I and JH II mainly regulate the growth and development of *Lepidoptera* larvae [3]. The isoprene-like branch synthesized by JH is the most studied. Studies have shown that in addition to certain *Hemiptera* insects, where corpora allata secrete a modified form called JH III, biepoxides are skipped [4]. In other insects, P450 enzymes are also involved in the synthesis of JH. For example, in *Diploptera punctata*, CYP4C7 can convert JH III to 12-trans-hydroxy JH III and metabolize other JH-like sesquiterpenoids [5]. CYP15A1 catalyzes the epoxidation of farnesol methyl ether to JH III in cockroach bodies [6]. Unfortunately, there is currently no more information on the direct effects of *BmFAMeT* on JH synthesis.

Initially, Meng et al. first identified seven homologous *BmFAMeT* genes in silkworms and cloned them [7]. Subsequently, our laboratory focused on *BmFAMeT6* and found that it is highly expressed during the first and second instar larvae stages of the Trimolter strain. Following interference treatment of the Trimolter strain with *BmFAMeT6*, the molting time of larvae increased, resulting in a transformation of the Trimolter strain into a tetramolter strain. Furthermore, immunofluorescence results indicated that the expression pattern of *BmFAMeT6* correlates with changes in JH [8]. Therefore, we hypothesize that *BmFAMeT6* influences JH levels, thereby affecting the molting of silkworm larvae. Using transgenic technology and high-performance liquid chromatography, we conducted overexpression and knockout experiments on *BmFAMeT6* in individual specimens and measured JH levels. The results repeatedly confirmed that *BmFAMeT6* influences the developmental stages of silkworm larvae by affecting JH titers [9]. However, the underlying mechanism by which *BmFAMeT6* affects JH levels remains under continuous investigation. The completion of the silkworm genome sequencing project in 2004 has provided important foundational data for research in related fields [10].

Therefore, this study, based on the existing overexpression strain of *BmFAMeT6*, utilizes transcriptomics and metabolomics approaches to gain a macroscopic understanding of the potential pathways through which *BmFAMeT6* may affect JH. It provided valuable information for a systematic understanding of the regulatory effects of *BmFAMeT6* on insect larval development and function.

## 2. Material and Methods

### 2.1. Insects and Environment

Larvea of B. mori (Dazao, BmFAMeT6 overexpression) were conserved in our laboratory [9], institute of college sericulture, textile, and biomass sciences, Southwest University, Beibei, China. The larvae were fed with fresh mulberry leaves at 12(L): 12(d) h photoperiod and 27 ± 1 °C. 

### 2.2. Experiment Design and Material Collection

In this study, the Dazao strain was used as a control group, while the Dazao strain overexpressing the *BmFAMeT6* was used as the experimental group. Two groups of bacterial strains were cultured to the third instar stage, with three replicates per group and 200 larvae per replicate.

We randomly selected larvae from two groups (control group and treatment group). Subsequently, larvae at the third instar stage were collected at 0, 12, and 24 h (C_Ov_0, C_Ov_12, C_Ov_24; Ov_0, Ov_12, Ov_24). To eliminate the influence of residual mulberry leaves, aseptic procedures were followed to remove the midgut. The entire process was conducted on ice. There were three replicates per group and three larvae per repeat.

### 2.3. Total RNA Isolation, Preparation, and Sequencing of Digital Gene Expression (DGE) Library

Total RNA was extracted from larvae using Trizol reagent (Takara, Dalian, China), and the RNA quality was evaluated on an Agilent 2100 Bioanalyzer (Agilent Technologies, Palo Alto, CA, USA). Subsequently, mRNA was enriched from the total RNA using Oligo(dT) beads. The enriched mRNA was then fragmented into short segments using a fragmentation buffer and reverse transcribed into complementary DNA (cDNA) using random primers. After RNA extraction, purification, and library construction, Next-Generation Sequencing (NGS) was performed on these libraries using the Illumina HiSeq sequencing platform in paired-end (PE) mode. The raw data were filtered to obtain high-quality sequences (Clean Data), which were then aligned to the silkworm genome database (https://silkbase.ab.a.u-tokyo.ac.jp/cgi-bin/news.cgi, accessed on 15 May 2023). Gene-expression levels were calculated based on the alignment results, followed by further analyses including differential expression analysis, enrichment analysis, and clustering analysis of the samples. To improve accuracy of differentially expressed genes (DEGs), we selected the genes with one-fold differences and Q-values <0.05 and defined them as significantly DEGs.

### 2.4. Metabolite Detection and Differential Metabolite Identification

Methods refer to Zhong and Deng et al., but with some modifications [11,12]. Samples (whole-body except midgut) from each group were centrifuged at 4 °C and 12,000 rpm for 10 min. The supernatant of each sample was transferred into a new 1.5 mL tube. After centrifugation, the samples were blow-dried by vacuum concentration. All of the dried samples were dissolved in 400 µL of a 2-chlorobenzalanine (0.0004%) methanol aqueous solution (1:1, 4 °C) and filtered with a 0.22 µm membrane. Then, the samples were ready for LC–MS. Chromatographic separation of the samples was accomplished in a Thermo Ultimate 3000 (Waltham, MA, USA) system equipped with an ACQUITY UPLC^®^ HSS T3 (150 × 2.1 mm, 1.8 µm, Waters, Milford, CT, USA) column maintained at 40 °C. At an autosampler temperature of 8 °C, gradient elution of the sample was carried out with 0.1% formic acid in water and 0.1% formic acid in acetonitrile at a flow rate of 0.25 mL min^−1^. Then, the separated samples were subjected to electrospray ionization tandem mass spectrometry experiments on a Thermo Q Exactive mass spectrometer (Waltham, MA, USA). The data-dependent acquisition Tandem mass spectrometry (MS/MS) system detected signals by high-energy collision-induced dissociation scanning. The original liquid chromatography–mass spectrometry data were finally obtained.

The original LC–MS data of metabolites in all samples were standardized and used for multivariate statistical analysis, including principal component analysis, partial least squares–discriminant analysis, and orthogonal projections to latent structures–discriminant analysis. Metabolite identification was initially confirmed based on accurate molecular weight. Subsequently, the metabolites were annotated using MS/MS fragmentation patterns against the Human Metabolome Database (HMDB) (http://www.hmdb.ca, accessed on 8 November 2022), MassBank (http://www.massbank.jp/, 10 November 2022), LipidMaps (http://www.lipidmaps.org, 15 November 2022), mzCloud (https://www.mzcloud.org, 20 November 2022). Differential metabolites were identified from the primary substance list of the samples, using the predetermined *p*-value (*p* < 0.05) and VIP threshold (VIP > 1.0) in the statistical analysis. 

### 2.5. Statistical Analysis of Data

All results were expressed as the mean ± SEM. of three biological replicates. Statistical differences were analyzed with Student’s *t*-test. Statistical difference levels were as follows, * (*p* < 0.05), ** (*p* < 0.01), and *** (*p* < 0.001).

## 3. Results

### 3.1. Overexpression of BmFAMeT6 Affected Gene Differential Expression in Silkworm Larvae

**Figure 1:**
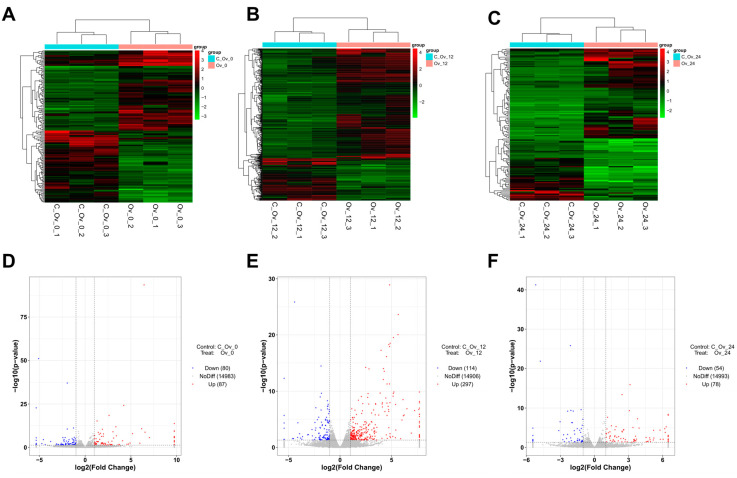
Expression difference analysis. (**A**), (**B**), and (**C**) The 3rd instar silkworm at 0 h, 12 h, and 24 h, respectively. Cluster analysis was performed based on the expression levels of the same gene in different samples and the expression patterns of different genes in the same sample. The Euclidean method was used to calculate distances, and hierarchical clustering was conducted using the Complete Linkage method. Red indicates high-expression genes, and green indicates low-expression genes. (**D**), (**E**), and (**F**) The 3rd instar silkworm at 0 h, 12 h, and 24 h, respectively. Volcano diagram: log2FoldChange in horizontal and −log10 (*p*-value) in vertical. The two vertical dotted lines are 2 times the difference threshold and the horizontal dotted lines are *p*-value = 0.05. Red dots indicate upregulated genes, blue dots indicate downregulated genes, and gray dots indicate non-significantly DEGs.

A cluster analysis heatmap revealed that the overexpressed treatment of *BmFAMeT6* resulted in DEGs in silkworms at 0, 12, and 24 h of the third instar stage. Compared to the control group, overexpression of *BmFAMeT6* resulted in 80, 114, and 54 upregulated genes, and 87, 297, and 78 downregulated genes at 0, 12, and 24 h of the third instar stage in silkworms, respectively. Obviously, the number of both upregulated and downregulated DEGs peaked at 12 h (Figure 1).

### 3.2. The Overexpression of BmFAMeT6 Mainly Affects the Molecular Functions and Biological Processes of Silkworm Larvae

**Figure 2:**
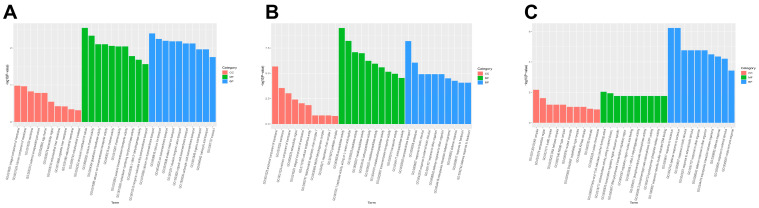
Gene Ontology (GO) enrichment analysis. (**A**), (**B**), and (**C**) The 3rd instar silkworm at 0 h, 12 h, and 24 h, respectively. Terms with GO level 2 level in horizontal coordinates and the −log10 (*p*-value) of each term in vertical coordinates. CC: cellular component; MF: molecular function; BP: biological process.

In the DEGs obtained from the comparison of two groups, Gene Ontology (GO) enrichment revealed that most of the DEGs were classified into three major functional categories: biological process (BP), cellular component (CC), and molecular function (MF). At 0 h and 12 h, the DEGs are primarily associated with molecular function (MF) and biological process (BP) categories. In the MF category, most of the DEGs at 0 h are enriched in structural constituent of cuticle (GO:0042302), calcium channel activity (GO:0005262), glutathione transferase activity (GO:0004364), and calcium ion transmembrane transporter activity (GO:0015085); while the DEGs at 12 h were primarily enriched in exopeptidase activity (GO:0008238), peptidase activity, acting on L-amino acid peptide (GO:0070011), and peptidase activity (GO:0008233). In the BP category, the DEGs were enriched in calcium ion transmembrane transport (GO:0070588), calcium ion transport (GO:0006816), ion transmembrane transport (GO:0034220), transmembrane transport (GO:0055085), proteolysis (GO:0006508), and response to biotic stimulus (GO:0009607) at 0 h and 12 h, respectively. Interestingly, the DEGs at 24 h were primarily enriched in biological process (BP) categories, including enrichment in response to bacterium (GO:0009671), defense response to bacterium (GO:0042742), and response to biotic stimulus (GO:0009607) (Figure 2).

### 3.3. BmFAMeT6 May Modulate Larval Metabolic Pathways through Zinc Finger Transcription Factors

**Figure 3:**
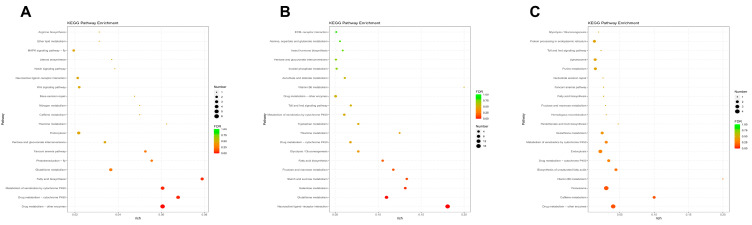
Enrichment pathway analysis. (**A**), (**B**), and (**C**) The 3rd instar silkworm at 0 h, 12 h, and 24 h, respectively. According to the GO enrichment results, the degree of enrichment was measured by Rich factor, FDR value, and the number of genes enriched on this GO Term. Rich factor is the ratio of the number of differentially enriched genes in the GO Term to the number of differentially annotated genes. The larger the Rich factor, the greater the enrichment. FDR values generally range from 0 to 1; the closer to zero, the more significant the enrichment.

Kyoto Encyclopedia of Genes and Genomes pathway classification and functional enrichment for DEGs were performed to identify the major biochemical metabolic pathways and signal-transduction pathways. As shown in the Figure 3, we plotted the top 20 enriched signaling pathways for the three time points. It is shown that at 0 h, the pathways primarily enriched include drug metabolism–other enzymes, drug metabolism–cytochrome P450, metabolism of xenobiotics by cytochrome P450, and fatty acid biosynthesis, while the DEGs enriched at 12 h are mainly associated with pathways neuroactive ligand–receptor interaction, glutathione metabolism, galactose metabolism, starch, and suctose metabolism (Figure 3A,B). At 24 h, the pathways primarily enriched are drug metabolism–other enzymes, caffeine metabolism, and peroxisome (Figure 3C). The specific DEGs were as shown in the table, and the repeated appearance of several DEGs (such as KWMTBOMO03670, KWMTBOMO03671) may be worth paying attention to (Table S1).

**Figure 4:**
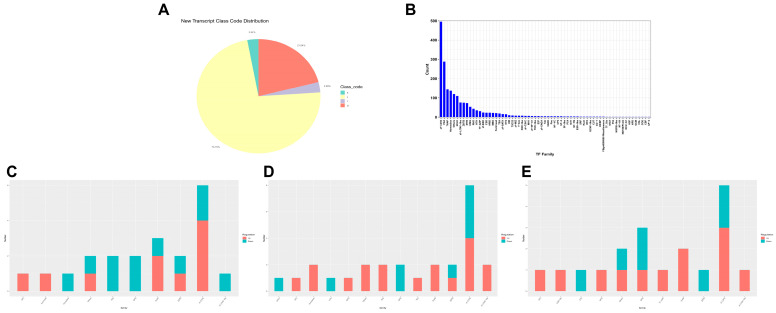
Structure and transcription factor family analysis. (**A**) New transcript analysis. X: Exon coverage on the anti-sense strand of known genes. J: Potential new transcripts (at least one variable splicing site shared with known transcripts). I: Transcript fragments completely within a known intron. U: Unknown segments (transcripts located in intergenic regions). (**B**) Transcription factor family diagram. The horizontal coordinate is different transcription factor family, the vertical coordinate is the number of genes falling into this transcription factor family. (**C**), (**D**), and (**E**) Histogram of differential transcription factors; the 3rd instar silkworm at 0 h, 12 h, and 24 h, respectively. The horizontal coordinate is a different transcription factor family; the vertical coordinate is the number of genes falling into this transcription factor family.

Further transcriptome statistical classification showed that 73.15% of the transcripts had potential new transcripts, with positional fragments accounting for 21.04%, exon coverage on the anti-sense strand of known genes and transcript fragments completely within a known intron accounting for 2.92% and 2.89%, respectively (Figure 4A). Transcription factor family statistics showed that the ZF-C2H2 transcription factor family dominates, followed by the PAX and THAP transcription factor families (Figure 4B). The number of DEGs (up: KWMTBOMO03663, KWMTBOMO16572, KWMTBOMO15898, KWMTBOMO06186; down: KWMTBOMO01816, KWMTBOMO06130, KWMTBOMO06133, KWMTBOMO14492) in the zf-C2H2 transcription factor family reaches its peak at 12 h (Figure 4C–E and Table S2). At 0, 12 and 24 h, the third instar larvae of silkworm, displayed the same differential genes: KWMTBOMO06130, KWMTBOMO03663 and KWMTBOMO06186 (Table S2). These transcription factors are closely related to gene expression, cell proliferation, differentiation and apoptosis, and organ development [13,14,15,16]. This suggested that overexpression *BmFAMeT6* has a certain connection to these biological processes in silkworms.

### 3.4. Overexpression of BmFAMeT6 Led to Metabolic Differences in the Silkworm Larvae

To further investigate the impact of *BmFAMeT6* overexpression on the growth and development of silkworms, this study continued transcriptome sequencing at three time points.

**Figure 5:**
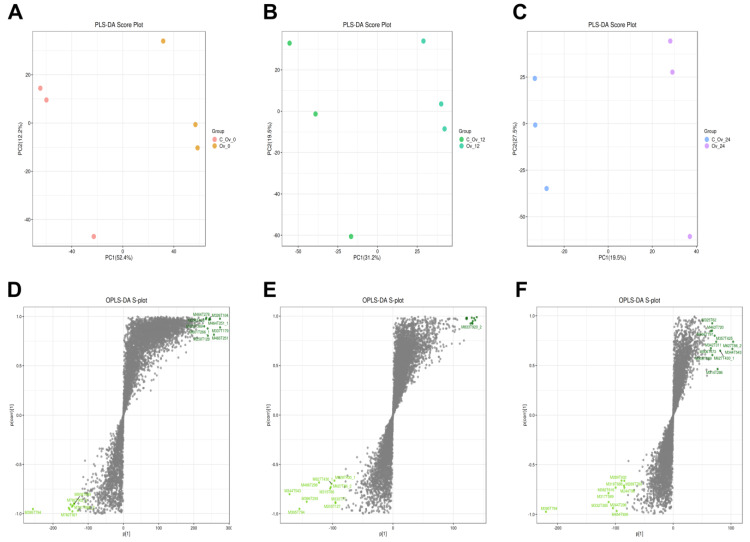
Multivariate statistical analysis. Negative ion mode. (**A**), (**B**), and (**C**) The 3rd instar silkworm at 0 h, 12 h, and 24 h, respectively. Partial least squares–discriminant analysis (PLS-DA). The first principal component interpretive degree is indicated by the horizontal coordinate, and the second principal component interpretive degree is indicated by the vertical coordinate. The dots represent the experimental samples, and the colors represent the different groups. The more clustered the intra-group samples and the more dispersed the inter-group samples were, the more reliable the results were. (**D**), (**E**), and (**F**) The 3rd instar silkworm at 0 h, 12 h, and 24 h, respectively. Orthogonal Projections to Latent Structures–Discriminant Analysis (OPLS-DA). The co-correlation coefficient between the principal component and the metabolite is shown on the horizontal coordinate, and the correlation coefficient between the principal component and the metabolite is shown on the vertical coordinate. The closer the two horns are, the more important the metabolites are.

A total of 541 metabolites were identified in this metabolomics analysis, of which 182 were significantly different. Partial least squares discriminant analysis (PLS-DA) showed that the samples were more clustered within each group, while the results were significantly dispersed between the experimental and control groups (Figure 5A–C). This indicated a close association between metabolic differences and the overexpression treatment.

### 3.5. The Differential Metabolites Caused by BmFAMeT6

**Figure 6:**
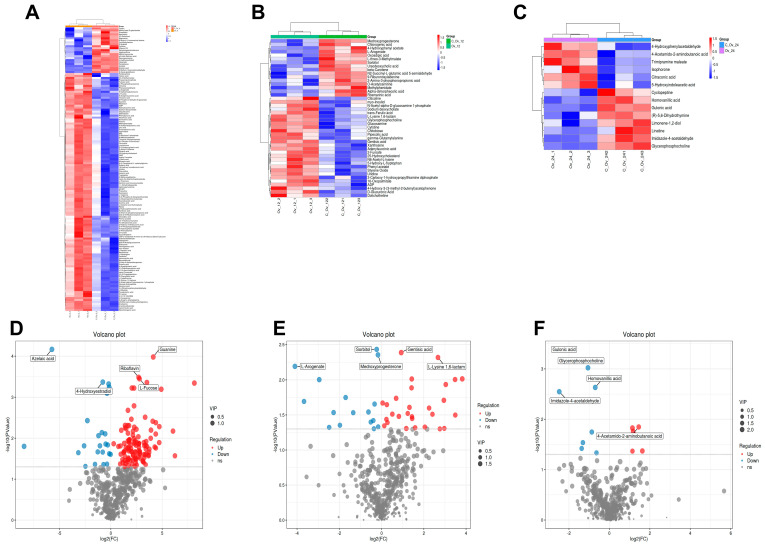
Identification of differential metabolites. (**A**), (**B**), and (**C**) The 3rd instar silkworm at 0 h, 12 h, and 24 h, respectively. Clustering hierarchical heatmap; the size of the relative content of the color shows the differences; the higher the blue color, the lower the expression level. The columns represent the samples, the rows represent the metabolite names, and the cluster tree on the left is the differential metabolite cluster tree. (**D)**, (**E**), and (**F**) The 3rd instar silkworm at 0 h, 12 h, and 24 h, respectively. Volcano map: each point in the graph represents a metabolite, with horizontal coordinates representing the log of Log2 for the multiple of quantitative difference for a metabolite in the two samples and vertical coordinates representing the log of −log10 for the *p* value. The larger the absolute value of the horizontal coordinate, the greater the fold difference of the expression amount of a metabolite between the two samples; the larger the vertical coordinate value, the more significant the differential expression, and the more reliable the screened differentially expressed metabolite is. Red dots represent upregulated differentially expressed metabolites, blue dots represent downregulated differentially expressed metabolites, and gray dots represent metabolites that were detected but did not meet the filter parameter screen.

Furthermore, the clustering heatmap showed that the numbers of differential metabolites at 0 h, 12 h, and 24 h in the third instar are 143, 44, and 14, respectively (Figure 6A–C). Using Orthogonal Projections to Latent Structures–Discriminant Analysis (OPLS-DA), we identified the top 10 metabolites (Figure 5D–F). Among related metabolites, the 0 h time point completely differed from the other two time points, while the 12 h and 24 h time points exhibited similar enriched metabolites (such as M344T543 (JWH 073 5-hydroxyindole metabolite)) (Figure 5D–F). These results indicated that *BmFAMeT6* continues to influence the metabolism of third instar silkworm larvae, and mulberry feeding may also affect metabolism. Continuing with the screening of differential metabolites, the top five differentially abundant metabolites at three time points were identified as Azelaic acid, guanine, riboflavin, 44-hydroxyestradiol, L-fucose (0 h); sorbitol, medroxyprogesterone, gentisic acid, L-lysine 1,6-lactam, L-arogenate (12 h); glulonic acid glycerophosphocholine, homovanillic acid, imidazole-4-acetaldehyde, 4-acetamldo-2-aminobutanoic acid (24 h) (Figure 6D–F). 

**Figure 7:**
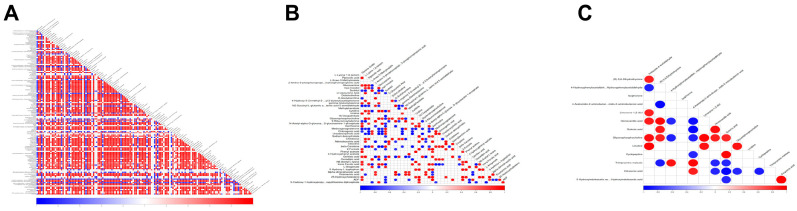
Differential metabolite association heatmap. (**A**), (**B**), and (**C**) The 3rd instar silkworm at 0 h, 12 h, and 24 h, respectively. Both the vertical and oblique vertical coordinates represent the names of the differential metabolites, and color represents correlation, with positive red correlation and negative blue correlation, and the darker the color, the greater the correlation.

A more intriguing finding emerged when calculating the Pearson correlation coefficients between all pairs of metabolites, revealing certain relationships among them (Figure 7A–C). For example, at 24 h of the third instar stage, gluonic acid and homovanillic acid, which are significantly decreased due to the overexpression of *BmFAMeT6* compared to the control, are positively correlated (Figure 7C). This suggests that the regulation of *BmFAMeT6* is also involved in the complex metabolic network of the silkworm.

### 3.6. The Main Metabolism Pathway and the Same Differential Metabolite by Overexpression of BmFAMeT6

**Figure 8:**
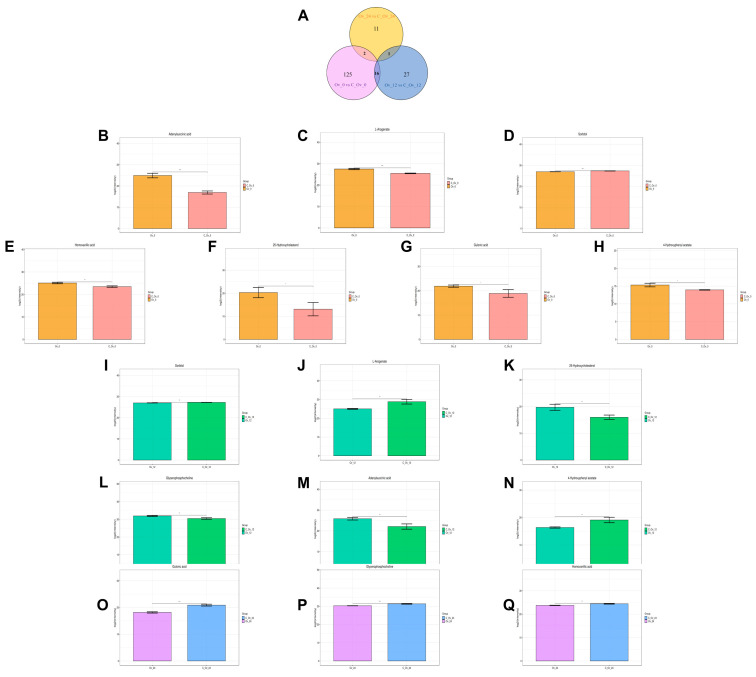
Comparison of differential metabolites. Histogram: horizontal coordinates for different groups; vertical coordinates for metabolite signal value range. Stars between the two groups indicate significant differences between the two groups, * *p* < 0.05, ** *p* < 0.01, *** *p* < 0.001, **** *p* < 0.0001. (**A**) Venn diagram: differences in metabolite counts at three time points (0 h, 12 h, 24 h). (**B**–**H**) The 3rd instar silkworm at 0 h; (**I**–**N**) the 3rd instar silkworm at 12 h; (**O**), (**P**), and (**Q**) the 3rd instar silkworm at 24 h.

In order to determine which metabolites are continuously affected by the overexpression of *BmFAMeT6*, we combined the differential metabolites from three time points and plotted a Venn diagram. We found that 16 identical metabolites were detected at 0 and 12 h, only 1 was detected at 0 and 24 h, and 2 were detected at 12 and 24 h (Figure 8A). Surprisingly, we found that these same differential metabolites were in part among the top 10 differential metabolites at the three time points, including adenylsuccinic acid, L-arogenate, sorbitol, 25-hydroxycholesterol, 4-hydroxyphenyl acetate, gulonic acid, homovanillic acid, and glycerophospholine (Figure 8B–Q). Furthermore, we observed that the trends in the changes of these three metabolites remained consistent across different time points, with sorbitol significantly decreased, 25-hydroxycholesterol significantly increased, and adenylsuccinic acid significantly increased (Figure 8B,D,F,I,K,M).

**Figure 9:**
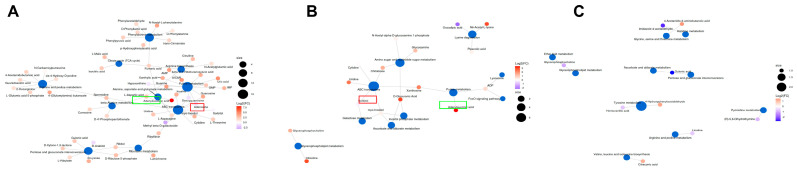
Analysis of differential metabolic pathways. (**A**–**C**) The 3rd instar silkworm at 0 h, 12 h, and 24 h. The blue dots represent pathways, while other dots represent metabolites. The size of the pathway dots indicates the number of connected metabolites, with larger dots representing more connections. The metabolite dots are color-coded according to the magnitude of their log2 (FC) values. However, it should be noted that in the case of multiple comparisons, the log2 (FC) information for metabolites is currently unavailable.

Then, in the metabolic pathway-enrichment analysis of the differential metabolites, we found that the main metabolic pathways involved included ABC transporters, purine metabolism, amino sugar and nucleotide sugar metabolism, and tyrosine metabolism, among others (Figure 9A–C). The important point is that among the three metabolites continuously affected (Figure 8), two are directly associated with the ABC transporters and purine metabolism (Figure 9A,B).

## 4. Discussion

### 4.1. The Regulatory Role of BmFAMeT6 Is Carried Out in the Context of Growth and Development

To explore the effect of *BmFAMeT6* overexpression on the transcription of growth- and development-related genes, DGE databases were established and analyzed for *BmFAMeT6* individuals at 0, 12, and 24 h of the third instar stage in silkworms. The results showed that 80, 114, 54 upregulated genes and 87, 297, 78 downregulated genes were obtained from *BmFAMeT6* overexpressed individuals at three time points of third age, respectively. The peak of DGEs at 12 h seems to indicate that the regulation of *BmFAMeT6* is a turning point at 12 h. However, in the data, the most significant feature was the enrichment of molecular functions related to the stratum corneum structure (GO: 0042302) at 0 h. The data at 12 h were mainly associated with the enrichment of molecular functions related to exopeptidase activity (GO:0008238). At 24 h, the focus was mainly on the enrichment of biological processes related to response to bacterium (GO:0009671). This change seems to reflect the biological processes with growth and development in silkworms, which is in line with exoskeleton sclerosis after just molting, nutrient digestion, and absorption, and against pathogens and toxic substances from mulberry leaves. Moreover, the types of metabolites (such as decanedioic acid, gluconic acid, glycerophosphorylcholine, etc.) in this study were found to be largely consistent with those identified in different developmental stages of silkworms in previous analyses [17], indicating that nutrient absorption gradually predominates in the life activities and metabolic processes of silkworms with time. In particular, the decreasing trend in the number of differential metabolites from 0 to 24 h suggested a gradual convergence of the regulatory role of *BmFAMeT6*. Additionally, receptor tyrosine kinases bind with prolactin and activate ecdysone synthesis through the MAPK/ERK (mitogen-activated protein kinase/extracellular signal-regulated kinase) pathway [18,19]. the most significant tyrosine metabolic pathway within 24 h of the third instar implies a more active synthesis process of ecdysteroids, which have an antagonistic relationship with JH. These indicated that the changes in silkworms induced by *BmFAMeT6* occur under the regulation of the silkworm’s own growth and development rhythm. 

In addition, L-fucose is a constituent of glycoconjugates in different organisms; during the growth and development of silkworms, there is a decrease in fucose content as they age [20,21]. In this study, among the top five differentially expressed metabolites, L-fucose was only observed at the 0 h time point and was absent in the subsequent time points. Furthermore, the enriched metabolites differed between 0 h and the other two time points. These may be attributed to before and after feeding leaves.

### 4.2. Differential Gene Enrichment in the Metabolic Pathway Indicates That BmFAMeT6 May Be Involved in the Balance between Growth and Detoxification Metabolism

The overexpression of *BmFAMeT6* affected hormone changes in silkworms [9]. Cytochrome P450 enzymes are crucial in hormone biosynthesis and metabolism [22]. Transcriptome analysis results showed that terms related to drug metabolism–other enzymes, drug metabolism–cytochrome P450, and metabolism of xenobiotics by cytochrome P450 were identified in all three time points of the third instar stage (0, 12, and 24 h). These pathways contain the same genes (KWMTBOMO03670, KWMTBOMO03671). Although there is currently no research showing a relationship between these two genes and hormone regulation, there is a balance between hormone homeostasis and detoxification metabolism [23]. We speculate that they may be related to hormone changes induced by overexpression of *BmFAMeT6*. 

### 4.3. The Zinc Finger C2H2 (zf-C2H2) Transcription Factor May Have Made an Important Contribution to the Regulation of BmFAMeT6

Analysis of transcription factors revealed that the zf-C2H2 family was dominant, and zinc finger protein 28-like (KWMTBOMO06130) was consistently downregulated at all three time points, while zinc finger protein 235-like (KWMTBOMO03663) and zinc finger protein 28-like (KWMTBOMO06186) were consistently upregulated. Zinc finger transcription factors are closely related to hormone regulation, cell development, limb growth, and gender differentiation in insects [24,25,26,27]. What is more, Kr-h 1 encodes a transcription factor with eight C2H2 zinc fingers and has been identified as a JH primary response gene activated by the JH receptor Met/Gce in various insect species [28,29]. Moreover, the third instar of silkworm larvae is the peak of juvenile hormone titers [3]. This study suggested that these zinc finger transcription factors may be closely related to the hormonal changes during the third instar larval stage, but further validation is required. 

### 4.4. The BmFAMeT6 May Influence the Metabolism of Silkworm Larvae by Participating in the ABC Transporters, Purine Metabolism Pathway, and Tyrosine Metabolism

ABC transporters play pivotal roles in a multitude of physiological processes, encompassing nutrient absorption, secretion of hormones, maintenance of lipid homeostasis, and detoxification metabolism [30]. Purine metabolism is crucial for the normal growth and development of silkworms, Fujii et al. used CRISPR/Cas9 technology to demonstrate that defects in purine metabolism leads to a translucent cuticle and male sterility in silkworm larvae [31]. In this study, it was found that sorbitol and adenosine succinate were directly associated with the ABC transporter and purine metabolism pathways, respectively (at 0 and 12 h), with sorbitol showing a decreasing trend at both time points and adenosine succinate showing an increasing trend. The decrease in sorbitol concentration is often accompanied by the progression of development [32]. Overexpression of *BmFAMeT6* leads to a decrease in JH levels and a shortened larval stage in silkworms [9]. It is suggested that *BmFAMeT6*, within the context of its growth and developmental regulation, might have an impact by engaging in ABC transporters and purine metabolism, which in turn would affect the metabolism of silkworm larvae.

## 5. Conclusions

In this study, we provided further evidence demonstrating that *BmFAMeT6* regulates the growth and development of silkworms during the third instar stage. Additionally, the regulatory role of *BmFAMeT6* occurs within the context of the silkworm’s own life processes, and the regulation of *BmFAMeT6* undergoes a turning point at the third instar 12 h with the changes in growth and development. The entire process involves zinc finger transcription factors, ABC transporters, purine metabolism, and tyrosine metabolism pathways. These findings contribute to our understanding of the regulatory network governing the growth and development of silkworms and insects.

## Data Availability

Data supporting the findings of this study are available to the corresponding authors.

## Supplementary Materials

The following supporting information can be downloaded at: https://www.mdpi.com/article/10.3390/genes15101261/s1, Table S1. Differential metabolic pathways of Top15 at three time points; Table S2. Differential transcription factors at three time points.
