# Supplementary material for: Transcriptome and Metabolome Analysis of BmFAMeT6 Overexpression in Bombyx mori
Source: Genes (Basel). 2024 Sep 27;15(10):1261. doi: 10.3390/genes15101261 (PMC11506984; doi:10.3390/genes15101261)
Supplement: Supplementary file 1 [file genes-15-01261-s001.zip › genes-3170180-supplementary.pdf]

Table S1. Differential metabolic pathways of Top15 at three time points

PathwayID	Pathway	DEG_number	Pvalue	FDR	Up_gene	Down_gene
**C_OV_0 VS OV_0**						
bmor00983	Drug metabolism - other enzymes	6	0.0001816	0.007084076	KWMTBOMO10692(KWMTBOMO10692),KWMTBOMO03670(KWMTBOMO03670),KWMTBOMO13242(KWMTBOMO13242),KWMTBOMO03671(KWMTBOMO03671)	KWMTBOMO02832(KWMTBOMO02832),KWMTBOMO15290(KWMTBOMO15290)
bmor00982	Drug metabolism - cytochrome P450	4	0.0016203	0.03159514	KWMTBOMO03670(KWMTBOMO03670),KWMTBOMO03671(KWMTBOMO03671),KWMTBOMO13242(KWMTBOMO13242)	KWMTBOMO15290(KWMTBOMO15290)
bmor00980	Metabolism of xenobiotics by cytochrome P450	4	0.002457	0.031941074	KWMTBOMO13242(KWMTBOMO13242),KWMTBOMO03671(KWMTBOMO03671),KWMTBOMO03670(KWMTBOMO03670)	KWMTBOMO15290(KWMTBOMO15290)
bmor00061	Fatty acid biosynthesis	3	0.0042752	0.041682805	KWMTBOMO09860(KWMTBOMO09860),KWMTBOMO09859(KWMTBOMO09859)	KWMTBOMO09857(KWMTBOMO09857)
bmor00480	Glutathione metabolism	3	0.0345399	0.242747315	KWMTBOMO03671(KWMTBOMO03671),KWMTBOMO13242(KWMTBOMO13242),KWMTBOMO03670(KWMTBOMO03670)	-
bmor04745	Phototransduction - fly	2	0.0394727	0.242747315	-	KWMTBOMO08349(KWMTBOMO08349),KWMTBOMO08347(KWMTBOMO08347)
bmor03460	Fanconi anemia pathway	2	0.04357	0.242747315	-	KWMTBOMO12376(KWMTBOMO12376),KWMTBOMO10036(KWMTBOMO10036)
bmor00040	Pentose and glucuronate interconversions	2	0.0946683	0.452975201	KWMTBOMO12375(KWMTBOMO12375)	KWMTBOMO15290(KWMTBOMO15290)
bmor04144	Endocytosis	3	0.120289	0.452975201	KWMTBOMO03667(KWMTBOMO03667),KWMTBOMO04434(KWMTBOMO04434)	KWMTBOMO16090(KWMTBOMO16090)
bmor00730	Thiamine metabolism	1	0.1325212	0.452975201	-	KWMTBOMO13810(KWMTBOMO13810)
bmor00232	Caffeine metabolism	1	0.1628936	0.452975201	KWMTBOMO10692(KWMTBOMO10692)	-
bmor00910	Nitrogen metabolism	1	0.1628936	0.452975201	KWMTBOMO03689(KWMTBOMO03689)	-
bmor03410	Base excision repair	1	0.1703242	0.452975201	-	KWMTBOMO16039(KWMTBOMO16039)
bmor04310	Wnt signaling pathway	2	0.1911082	0.452975201	-	KWMTBOMO12435(KWMTBOMO12435),KWMTBOMO11247(KWMTBOMO11247)
bmor04080	Neuroactive ligand-receptor interaction	2	0.2007938	0.452975201	KWMTBOMO02953(KWMTBOMO02953)	KWMTBOMO12573(KWMTBOMO12573)
**C_OV_12 VS OV_12**						
bmor04080	Neuroactive ligand-receptor interaction	17	5.046E-11	3.43123E-09	KWMTBOMO03768(KWMTBOMO03768),KWMTBOMO08714(KWMTBOMO08714),KWMTBOMO03771(KWMTBOMO03771),KWMTBOMO02955(KWMTBOMO02955),KWMTBOMO12647(KWMTBOMO12647),KWMTBOMO04031(KWMTBOMO04031),KWMTBOMO03769(KWMTBOMO03769),KWMTBOMO15920(KWMTBOMO15920),KWMTBOMO02953(KWMTBOMO02953),KWMTBOMO02956(KWMTBOMO02956),KWMTBOMO04764(KWMTBOMO04764),KWMTBOMO03772(KWMTBOMO03772),KWMTBOMO03770(KWMTBOMO03770),KWMTBOMO04766(KWMTBOMO04766),KWMTBOMO15917(KWMTBOMO15917),KWMTBOMO00946(KWMTBOMO00946),KWMTBOMO03767(KWMTBOMO03767)	-
bmor00480	Glutathione metabolism	9	0.0001585	0.005388643	KWMTBOMO04888(KWMTBOMO04888),KWMTBOMO03671(KWMTBOMO03671),KWMTBOMO04889(KWMTBOMO04889),KWMTBOMO04891(KWMTBOMO04891),KWMTBOMO03670(KWMTBOMO03670),KWMTBOMO04892(KWMTBOMO04892),KWMTBOMO04887(KWMTBOMO04887),KWMTBOMO04900(KWMTBOMO04900)	KWMTBOMO03300(KWMTBOMO03300)
bmor00052	Galactose metabolism	5	0.0022177	0.050267921	KWMTBOMO16620(KWMTBOMO16620),KWMTBOMO07101(KWMTBOMO07101),KWMTBOMO01629(KWMTBOMO01629),KWMTBOMO07102(KWMTBOMO07102),KWMTBOMO01630(KWMTBOMO01630)	-
bmor00500	Starch and sucrose metabolism	4	0.0059431	0.101033256	KWMTBOMO01629(KWMTBOMO01629),KWMTBOMO07450(KWMTBOMO07450),KWMTBOMO01630(KWMTBOMO01630),KWMTBOMO16620(KWMTBOMO16620)	-
bmor00051	Fructose and mannose metabolism	4	0.009327	0.126847226	KWMTBOMO12375(KWMTBOMO12375),KWMTBOMO02882(KWMTBOMO02882),KWMTBOMO12374(KWMTBOMO12374),KWMTBOMO16620(KWMTBOMO16620)	-
bmor00061	Fatty acid biosynthesis	4	0.0137706	0.156066294	KWMTBOMO09851(KWMTBOMO09851),KWMTBOMO09860(KWMTBOMO09860),KWMTBOMO09859(KWMTBOMO09859)	KWMTBOMO09857(KWMTBOMO09857)
bmor00010	Glycolysis / Gluconeogenesis	4	0.038893	0.37781805	KWMTBOMO04269(KWMTBOMO04269),KWMTBOMO12093(KWMTBOMO12093),KWMTBOMO16620(KWMTBOMO16620)	KWMTBOMO11356(KWMTBOMO11356)
bmor00982	Drug metabolism - cytochrome P450	4	0.0574414	0.441113495	KWMTBOMO03670(KWMTBOMO03670),KWMTBOMO10971(KWMTBOMO10971),KWMTBOMO03671(KWMTBOMO03671)	KWMTBOMO03300(KWMTBOMO03300)
bmor00730	Thiamine metabolism	2	0.0583827	0.441113495	KWMTBOMO01603(KWMTBOMO01603),KWMTBOMO01604(KWMTBOMO01604)	-
bmor00380	Tryptophan metabolism	3	0.0712226	0.484313359	KWMTBOMO12792(KWMTBOMO12792)	KWMTBOMO02034(KWMTBOMO02034),KWMTBOMO11356(KWMTBOMO11356)
bmor00980	Metabolism of xenobiotics by cytochrome P450	4	0.079974	0.494384767	KWMTBOMO03670(KWMTBOMO03670),KWMTBOMO10971(KWMTBOMO10971),KWMTBOMO03671(KWMTBOMO03671)	KWMTBOMO03300(KWMTBOMO03300)
bmor04624	Toll and Imd signaling pathway	3	0.0946144	0.512177021	KWMTBOMO04838(KWMTBOMO04838),KWMTBOMO04967(KWMTBOMO04967),KWMTBOMO16532(KWMTBOMO16532)	-
bmor00983	Drug metabolism - other enzymes	5	0.0979162	0.512177021	KWMTBOMO03671(KWMTBOMO03671),KWMTBOMO10971(KWMTBOMO10971),KWMTBOMO03670(KWMTBOMO03670)	KWMTBOMO10808(KWMTBOMO10808),KWMTBOMO03300(KWMTBOMO03300)
bmor00750	Vitamin B6 metabolism	1	0.118187	0.547279184	-	KWMTBOMO09750(KWMTBOMO09750)
bmor00053	Ascorbate and aldarate metabolism	3	0.1207233	0.547279184	KWMTBOMO10971(KWMTBOMO10971),KWMTBOMO09531(KWMTBOMO09531)	KWMTBOMO11356(KWMTBOMO11356)
**C_OV_24 VS OV_24**						
bmor00983	Drug metabolism - other enzymes	4	0.0042598	0.121151293	KWMTBOMO10695(KWMTBOMO10695),KWMTBOMO10696(KWMTBOMO10696),KWMTBOMO03671(KWMTBOMO03671),KWMTBOMO03670(KWMTBOMO03670)	-
bmor00232	Caffeine metabolism	2	0.0080417	0.121151293	KWMTBOMO10695(KWMTBOMO10695),KWMTBOMO10696(KWMTBOMO10696)	-
bmor04146	Peroxisome	4	0.0117243	0.121151293	KWMTBOMO00252(KWMTBOMO00252),KWMTBOMO00253(KWMTBOMO00253),KWMTBOMO10695(KWMTBOMO10695),KWMTBOMO10696(KWMTBOMO10696)	-
bmor00750	Vitamin B6 metabolism	1	0.0340289	0.233905435	-	KWMTBOMO09750(KWMTBOMO09750)
bmor01040	Biosynthesis of unsaturated fatty acids	2	0.0377267	0.233905435	KWMTBOMO03331(KWMTBOMO03331)	KWMTBOMO07359(KWMTBOMO07359)
bmor00982	Drug metabolism - cytochrome P450	2	0.0615063	0.290233192	KWMTBOMO03671(KWMTBOMO03671),KWMTBOMO03670(KWMTBOMO03670)	-
bmor04144	Endocytosis	3	0.0673877	0.290233192	KWMTBOMO04434(KWMTBOMO04434),KWMTBOMO03667(KWMTBOMO03667),KWMTBOMO16090(KWMTBOMO16090)	-
bmor00980	Metabolism of xenobiotics by cytochrome P450	2	0.0748989	0.290233192	KWMTBOMO03671(KWMTBOMO03671),KWMTBOMO03670(KWMTBOMO03670)	-
bmor00480	Glutathione metabolism	2	0.1085421	0.373867256	KWMTBOMO03670(KWMTBOMO03670),KWMTBOMO03671(KWMTBOMO03671)	-
bmor00770	Pantothenate and CoA biosynthesis	1	0.1356116	0.420396102	-	KWMTBOMO16194(KWMTBOMO16194)
bmor03440	Homologous recombination	1	0.2049833	0.447798525	-	KWMTBOMO12376(KWMTBOMO12376)
bmor00051	Fructose and mannose metabolism	1	0.2105166	0.447798525	KWMTBOMO12375(KWMTBOMO12375)	-
bmor00061	Fatty acid biosynthesis	1	0.2322821	0.447798525	KWMTBOMO09851(KWMTBOMO09851)	-
bmor03460	Fanconi anemia pathway	1	0.2322821	0.447798525	-	KWMTBOMO12376(KWMTBOMO12376)
bmor03420	Nucleotide excision repair	1	0.2376328	0.447798525	KWMTBOMO16531(KWMTBOMO16531)	-
bmor00230	Purine metabolism	2	0.2459789	0.447798525	KWMTBOMO10696(KWMTBOMO10696),KWMTBOMO10695(KWMTBOMO10695)	-
bmor03040	Spliceosome	2	0.2537534	0.447798525	KWMTBOMO16524(KWMTBOMO16524),KWMTBOMO16090(KWMTBOMO16090)	-
bmor04624	Toll and Imd signaling pathway	1	0.2638537	0.447798525	KWMTBOMO16532(KWMTBOMO16532)	-
bmor04141	Protein processing in endoplasmic reticulum	2	0.2849312	0.447798525	KWMTBOMO16090(KWMTBOMO16090)	KWMTBOMO14275(KWMTBOMO14275)
bmor00010	Glycolysis / Gluconeogenesis	1	0.3040153	0.447798525	KWMTBOMO12093(KWMTBOMO12093)	-
bmor00020	Citrate cycle (TCA cycle)	1	0.3088851	0.447798525	KWMTBOMO12093(KWMTBOMO12093)	-
bmor00620	Pyruvate metabolism	1	0.3185266	0.447798525	KWMTBOMO12093(KWMTBOMO12093)	-
bmor00040	Pentose and glucuronate interconversions	1	0.3374238	0.447798525	KWMTBOMO12375(KWMTBOMO12375)	-
bmor00260	Glycine, serine and threonine metabolism	1	0.3466827	0.447798525	KWMTBOMO07027(KWMTBOMO07027)	-
bmor04391	Hippo signaling pathway - fly	1	0.4081035	0.506048379	KWMTBOMO16578(KWMTBOMO16578)	-
bmor04213	Longevity regulating pathway - multiple species	1	0.4789656	0.538421788	KWMTBOMO16090(KWMTBOMO16090)	-
bmor04080	Neuroactive ligand-receptor interaction	1	0.4826536	0.538421788	KWMTBOMO02953(KWMTBOMO02953)	-
bmor03013	Nucleocytoplasmic transport	1	0.4863165	0.538421788	KWMTBOMO03022(KWMTBOMO03022)	-
bmor04013	MAPK signaling pathway - fly	1	0.5147365	0.550235619	KWMTBOMO16532(KWMTBOMO16532)	-
bmor04140	Autophagy - animal	1	0.5640075	0.582807792	-	KWMTBOMO14275(KWMTBOMO14275)
bmor04068	FoxO signaling pathway	1	0.8068708	0.806870834	KWMTBOMO12093(KWMTBOMO12093)	-

Table S2. Differential transcription factors at three time points.

Gene_ID	Symbol	Family	Description	Regulation
**C_Ov_0_vs_Ov_0**				
KWMTBOMO10648	KWMTBOMO10648	zf-C2H2	gi|34099638|gb|AAQ57129.1| endonuclease and reverse transcriptase-like protein [Bombyx mori]	Up Regulation
KWMTBOMO12462	KWMTBOMO12462	Others	gi|827554565|ref|XP_012548837.1| PREDICTED: protein ENL isoform X1 [Bombyx mori]	Down Regulation
KWMTBOMO06130	KWMTBOMO06130	zf-C2H2	gi|512889034|ref|XP_004922185.1| PREDICTED: zinc finger protein 28-like [Bombyx mori]	Down Regulation
KWMTBOMO06185	KWMTBOMO06185	ZBTB	gi|827537961|ref|XP_012548992.1| PREDICTED: zinc finger protein 28-like [Bombyx mori]	Up Regulation
KWMTBOMO03663	KWMTBOMO03663	zf-C2H2	gi|827548360|ref|XP_012546399.1| PREDICTED: zinc finger protein 235-like [Bombyx mori]	Up Regulation
KWMTBOMO04133	KWMTBOMO04133	ZBTB	gi|827546097|ref|XP_012545516.1| PREDICTED: zinc finger and BTB domain-containing protein 14 [Bombyx mori]	Down Regulation
KWMTBOMO03023	KWMTBOMO03023	THAP	gi|827555772|ref|XP_012549314.1| PREDICTED: piggyBac transposable element-derived protein 4-like isoform X3 [Bombyx mori]	Up Regulation
KWMTBOMO08233	KWMTBOMO08233	THAP	gi|827563851|ref|XP_012552435.1| PREDICTED: piggyBac transposable element-derived protein 3-like, partial [Bombyx mori]	Down Regulation
KWMTBOMO12435	KWMTBOMO12435	zf-LITAF-like	gi|827545742|ref|XP_012545365.1| PREDICTED: protein groucho-like isoform X3 [Bombyx mori]	Down Regulation
KWMTBOMO00115	KWMTBOMO00115	RHD	gi|829569577|ref|NP_001296525.1| transient receptor potential cation channel subfamily A member 1 [Bombyx mori] gi|591288361|dbj|BAO53207.1| transient receptor potential A1 [Bombyx mori] gi|591288369|dbj|BAO53211.1| transient receptor potential A1 [Bombyx mori]	Down Regulation
KWMTBOMO00118	KWMTBOMO00118	RHD	gi|829569577|ref|NP_001296525.1| transient receptor potential cation channel subfamily A member 1 [Bombyx mori] gi|591288361|dbj|BAO53207.1| transient receptor potential A1 [Bombyx mori] gi|591288369|dbj|BAO53211.1| transient receptor potential A1 [Bombyx mori]	Down Regulation
KWMTBOMO11175	KWMTBOMO11175	Others	gi|512909787|ref|XP_004927006.1| PREDICTED: muskelin [Bombyx mori]	Up Regulation
KWMTBOMO16514	KWMTBOMO16514	CP2	gi|512935834|ref|XP_004933371.1| PREDICTED: transcription factor CP2-like protein 1 isoform X3 [Bombyx mori]	Up Regulation
KWMTBOMO01693	KWMTBOMO01693	Homeobox	gi|827553295|ref|XP_004929335.2| PREDICTED: uncharacterized protein LOC100862767 [Bombyx mori]	Down Regulation
KWMTBOMO00395	KWMTBOMO00395	zf-C2H2	gi|827554732|ref|XP_012548904.1| PREDICTED: Krueppel-like factor 5 [Bombyx mori]	Up Regulation
KWMTBOMO03021	KWMTBOMO03021	THAP	gi|827555774|ref|XP_012549315.1| PREDICTED: piggyBac transposable element-derived protein 4-like isoform X4 [Bombyx mori]	Up Regulation
KWMTBOMO02947	KWMTBOMO02947	PAX	gi|22770470|gb|AAN06610.1|AF461149_1 transposase [Bombyx mori]	Down Regulation
KWMTBOMO09370	KWMTBOMO09370	PAX	gi|22770470|gb|AAN06610.1|AF461149_1 transposase [Bombyx mori]	Down Regulation
KWMTBOMO06186	KWMTBOMO06186	zf-C2H2	gi|827537961|ref|XP_012548992.1| PREDICTED: zinc finger protein 28-like [Bombyx mori]	Up Regulation
KWMTBOMO15311	KWMTBOMO15311	zf-C2H2	gi|642949720|emb|CCH14900.1| endonuclease-reverse transcriptase [Blattella germanica]	Down Regulation
KWMTBOMO07613	KWMTBOMO07613	Fork head	gi|827550224|ref|XP_004927987.2| PREDICTED: fork head domain transcription factor slp2 [Bombyx mori]	Up Regulation
**C_Ov_12_vs_Ov_12**				
KWMTBOMO16531	KWMTBOMO16531	MYB	gi|512887754|ref|XP_004921975.1| PREDICTED: DNA excision repair protein ERCC-6-like [Bombyx mori]	Up Regulation
KWMTBOMO16525	KWMTBOMO16525	zf-LITAF-like	gi|512887719|ref|XP_004921969.1| PREDICTED: U3 small nucleolar RNA-interacting protein 2 [Bombyx mori]	Up Regulation
KWMTBOMO03769	KWMTBOMO03769	PAX	gi|512893869|ref|XP_004923289.1| PREDICTED: trypsin, alkaline A-like [Bombyx mori]	Up Regulation
KWMTBOMO04133	KWMTBOMO04133	ZBTB	gi|827546097|ref|XP_012545516.1| PREDICTED: zinc finger and BTB domain-containing protein 14 [Bombyx mori]	Down Regulation
KWMTBOMO03663	KWMTBOMO03663	zf-C2H2	gi|827548360|ref|XP_012546399.1| PREDICTED: zinc finger protein 235-like [Bombyx mori]	Up Regulation
KWMTBOMO16572	KWMTBOMO16572	zf-C2H2	gi|512888119|ref|XP_004922036.1| PREDICTED: zinc finger protein 567-like isoform X1 [Bombyx mori]	Up Regulation
KWMTBOMO01816	KWMTBOMO01816	zf-C2H2	gi|298204337|gb|ADI61817.1| endonuclease-reverse transcriptase [Bombyx mori]	Down Regulation
KWMTBOMO06185	KWMTBOMO06185	ZBTB	gi|827537961|ref|XP_012548992.1| PREDICTED: zinc finger protein 28-like [Bombyx mori]	Up Regulation

KWMTBOMO11640	KWMTBOMO11640	PAX	gi|512890028|ref|XP_004922435.1| PREDICTED: classical arabinogalactan protein 9-like [Bombyx mori]	Up Regulation
KWMTBOMO06130	KWMTBOMO06130	zf-C2H2	gi|512889034|ref|XP_004922185.1| PREDICTED: zinc finger protein 28-like [Bombyx mori]	Down Regulation
KWMTBOMO00457	KWMTBOMO00457	HTH	gi|943940663|ref|XP_014371805.1| PREDICTED: uncharacterized protein LOC106721395 [Papilio machaon]	Down Regulation
KWMTBOMO15898	KWMTBOMO15898	zf-C2H2	gi|827540675|ref|XP_004923618.2| PREDICTED: sporozoite surface protein 2-like [Bombyx mori]	Up Regulation
KWMTBOMO05916	KWMTBOMO05916	TEA	gi|827560356|ref|XP_004932441.2| PREDICTED: leukocyte surface antigen CD53-like [Bombyx mori]	Up Regulation
KWMTBOMO11175	KWMTBOMO11175	Others	gi|512909787|ref|XP_004927006.1| PREDICTED: muskelin [Bombyx mori]	Up Regulation
KWMTBOMO07910	KWMTBOMO07910	Homeobox	gi|112984380|ref|NP_001037514.1| caudal-like [Bombyx mori] gi|521229|dbj|BAA04086.1| cad protein [Bombyx mori]	Up Regulation
KWMTBOMO03023	KWMTBOMO03023	THAP	gi|827555772|ref|XP_012549314.1| PREDICTED: piggyBac transposable element-derived protein 4-like isoform X3 [Bombyx mori]	Up Regulation
KWMTBOMO00118	KWMTBOMO00118	RHD	gi|829569577|ref|NP_001296525.1| transient receptor potential cation channel subfamily A member 1 [Bombyx mori] gi|591288361|dbj|BAO53207.1| transient receptor potential A1 [Bombyx mori] gi|591288369|dbj|BAO53211.1| transient receptor potential A1 [Bombyx mori]	Down Regulation
KWMTBOMO07115	KWMTBOMO07115	Homeobox	gi|148298661|ref|NP_001091838.1| pituitary homeobox1 [Bombyx mori] gi|121582239|dbj|BAF44479.1| pituitary homeobox1 [Bombyx mori]	Up Regulation
KWMTBOMO00115	KWMTBOMO00115	RHD	gi|829569577|ref|NP_001296525.1| transient receptor potential cation channel subfamily A member 1 [Bombyx mori] gi|591288361|dbj|BAO53207.1| transient receptor potential A1 [Bombyx mori] gi|591288369|dbj|BAO53211.1| transient receptor potential A1 [Bombyx mori]	Down Regulation
KWMTBOMO08307	KWMTBOMO08307	Others	gi|827538562|ref|XP_012551443.1| PREDICTED: cAMP-responsive element-binding protein-like 2 isoform X2 [Bombyx mori]	Up Regulation
KWMTBOMO16514	KWMTBOMO16514	CP2	gi|512935834|ref|XP_004933371.1| PREDICTED: transcription factor CP2-like protein 1 isoform X3 [Bombyx mori]	Up Regulation
KWMTBOMO06133	KWMTBOMO06133	zf-C2H2	gi|827537665|ref|XP_012548028.1| PREDICTED: zinc finger protein 271-like isoform X2 [Bombyx mori]	Down Regulation
KWMTBOMO15161	KWMTBOMO15161	zf-LITAF-like	gi|512898965|ref|XP_004924527.1| PREDICTED: lipopolysaccharide-induced tumor necrosis factor-alpha factor homolog [Bombyx mori]	Up Regulation
KWMTBOMO06186	KWMTBOMO06186	zf-C2H2	gi|827537961|ref|XP_012548992.1| PREDICTED: zinc finger protein 28-like [Bombyx mori]	Up Regulation
KWMTBOMO01408	KWMTBOMO01408	bHLH	gi|283483973|ref|NP_001164466.1| enhancer of split mbeta [Bombyx mori] gi|281428763|gb|ADA69993.1| E(spl)-like protein [Bombyx mori] gi|827884114|dbj|BAR88293.1| Enhancer of split mbeta [Bombyx mori]	Down Regulation
KWMTBOMO02948	KWMTBOMO02948	THAP	gi|943979160|ref|XP_014370475.1| PREDICTED: uncharacterized protein LOC106720343 [Papilio machaon]	Up Regulation
KWMTBOMO14492	KWMTBOMO14492	zf-C2H2	gi|298204339|gb|ADI61818.1| endonuclease-reverse transcriptase [Bombyx mori]	Down Regulation
**C_Ov_24_vs_Ov_24**				
KWMTBOMO04133	KWMTBOMO04133	ZBTB	gi|827546097|ref|XP_012545516.1| PREDICTED: zinc finger and BTB domain-containing protein 14 [Bombyx mori]	Down Regulation
KWMTBOMO06085	KWMTBOMO06085	zf-C2H2	gi|827537912|ref|XP_012548823.1| PREDICTED: zinc finger protein 660-like [Bombyx mori]	Down Regulation
KWMTBOMO11175	KWMTBOMO11175	Others	gi|512909787|ref|XP_004927006.1| PREDICTED: muskelin [Bombyx mori]	Up Regulation
KWMTBOMO12462	KWMTBOMO12462	Others	gi|827554565|ref|XP_012548837.1| PREDICTED: protein ENL isoform X1 [Bombyx mori]	Down Regulation
KWMTBOMO00118	KWMTBOMO00118	RHD	gi|829569577|ref|NP_001296525.1| transient receptor potential cation channel subfamily A member 1 [Bombyx mori] gi|591288361|dbj|BAO53207.1| transient receptor potential A1 [Bombyx mori] gi|591288369|dbj|BAO53211.1| transient receptor potential A1 [Bombyx mori]	Down Regulation
KWMTBOMO03023	KWMTBOMO03023	THAP	gi|827555772|ref|XP_012549314.1| PREDICTED: piggyBac transposable element-derived protein 4-like isoform X3 [Bombyx mori]	Up Regulation
KWMTBOMO15297	KWMTBOMO15297	RHD	gi|827536984|ref|XP_012545379.1| PREDICTED: ankyrin repeat domain-containing protein 12 [Bombyx mori]	Up Regulation
KWMTBOMO16525	KWMTBOMO16525	zf-LITAF-like	gi|512887719|ref|XP_004921969.1| PREDICTED: U3 small nucleolar RNA-interacting protein 2 [Bombyx mori]	Up Regulation
KWMTBOMO06130	KWMTBOMO06130	zf-C2H2	gi|512889034|ref|XP_004922185.1| PREDICTED: zinc finger protein 28-like [Bombyx mori]	Down Regulation
KWMTBOMO06186	KWMTBOMO06186	zf-C2H2	gi|827537961|ref|XP_012548992.1| PREDICTED: zinc finger protein 28-like [Bombyx mori]	Up Regulation
KWMTBOMO14514	KWMTBOMO14514	THAP	gi|913300085|ref|XP_013198796.1| PREDICTED: uncharacterized protein LOC106141703 [Amyelois transitella]	Up Regulation
KWMTBOMO03663	KWMTBOMO03663	zf-C2H2	gi|827548360|ref|XP_012546399.1| PREDICTED: zinc finger protein 235-like [Bombyx mori]	Up Regulation
KWMTBOMO16572	KWMTBOMO16572	zf-C2H2	gi|512888119|ref|XP_004922036.1| PREDICTED: zinc finger protein 567-like isoform X1 [Bombyx mori]	Up Regulation
KWMTBOMO00595	KWMTBOMO00595	ETS	gi|827562023|ref|XP_012551739.1| PREDICTED: uncharacterized protein LOC105842554 [Bombyx mori]	Down Regulation
KWMTBOMO16531	KWMTBOMO16531	MYB	gi|512887754|ref|XP_004921975.1| PREDICTED: DNA excision repair protein ERCC-6-like [Bombyx mori]	Up Regulation
KWMTBOMO16514	KWMTBOMO16514	CP2	gi|512935834|ref|XP_004933371.1| PREDICTED: transcription factor CP2-like protein 1 isoform X3 [Bombyx mori]	Up Regulation
KWMTBOMO14015	KWMTBOMO14015	RHD	gi|512925000|ref|XP_004930731.1| PREDICTED: ankyrin-3-like [Bombyx mori]	Down Regulation
KWMTBOMO03291	KWMTBOMO03291	ESR-like	gi|926614085|ref|XP_013772075.1| PREDICTED: probable nuclear hormone receptor HR3 [Limulus polyphemus]	Up Regulation
KWMTBOMO00330	KWMTBOMO00330	TF_bZIP	gi|827555190|ref|XP_012549087.1| PREDICTED: uncharacterized protein LOC101744279 isoform X2 [Bombyx mori]	Up Regulation
